# Perspectives on Clinical Champions Implementing Hospital-Based Opioid Treatment in US Hospitals

**DOI:** 10.1001/jamanetworkopen.2026.0446

**Published:** 2026-03-03

**Authors:** Linda Peng, Amelia Goff, Alisa Patten, Angela R. Bazzi, Carla King, Tracy Siegler, Zoe Weinstein, Riley Shearer, Hildi Hagedorn, Emily Oot, Gavin Bart, Udi E. Ghitza, Honora Englander

**Affiliations:** 1 Department of Medicine, Oregon Health and Science University, Portland; 2 Herbert Wertheim School of Public Health, University of California San Diego, La Jolla; 3 Boston University School of Public Health (Department of Community Health Sciences), Boston, Massachusetts; 4 Department of Population Health, New York University Grossman School of Medicine; 5 Center of Health Enhancement Systems Studies, University of Wisconsin-Madison; 6 Boston Medical Center and the Boston University Chobanian and Avedisian School of Medicine, Boston, Massachusetts; 7 University of Minnesota School of Public Health, Minneapolis; 8 Minneapolis Veterans Affairs Health Care System, Minneapolis, Minnesota; 9 University of Minnesota Medical School, Minneapolis; 10 Boston University, Boston, Massachusetts; 11 Hennepin Healthcare, Minneapolis, Minnesota; 12 National Institute on Drug Abuse, North Bethesda, Maryland

## Abstract

**Importance:**

Hospital-based opioid treatment (HBOT) can improve outcomes for patients with opioid use disorder, but little is known about specific attributes and supports needed for clinical champions to successfully implement HBOT.

**Objective:**

To identify characteristics and supports of effective clinical champions implementing HBOT in US hospitals.

**Design, Setting, and Participants:**

Qualitative study using postimplementation semistructured interviews conducted with individuals highly involved in HBOT implementation, including champions and hospital staff at 12 US community hospitals randomized to the high-intensity group of the Exemplar Hospital Initiation Trial to Enhance Treatment Engagement, a national implementation trial comparing low- and high-intensity HBOT implementation strategies. Interviews explored implementation experiences over 24 months from December 2021 to December 2023. Interviews were audio recorded, transcribed, and coded. The framework method and in-depth thematic analysis were used to explore the role of champions.

**Interventions:**

All hospitals received a best-practices manual, video webinar series, and hub team support for questions, while hospitals randomized to the high-intensity group also received monthly practice facilitation, telementoring, and 10% effort funding for a local champion. Champions led HBOT implementation with support from regional hubs with HBOT expertise.

**Measures:**

Effective champions were defined as those perceived by staff to successfully lead HBOT implementation. Open-ended questions and thematic analysis explored participants’ perspectives on attributes of effective champions and how they overcame implementation barriers.

**Results:**

A total of 31 hospital staff (15 physicians, 5 executives, 5 pharmacists, 2 nurse practitioners, 2 social workers, 1 nurse, and 1 addiction counselor) were interviewed. Effective champions were perceived as respected hospital “insiders” with institutional influence, persistence, and systems change skills. They built multidisciplinary teams, developed standard workflows, and used emotionally resonant strategies (eg, patient narratives) to overcome stigma and engage hospital leadership. Champions could be effective without addiction medicine expertise, particularly when provided with protected time, hospital leadership support, and external practice facilitation from addiction experts.

**Conclusions and Relevance:**

This multisite qualitative study underscores the vital role of champions in expanding hospital-based opioid care. To ensure HBOT expansion, hospitals should invest in champions, protected time, leadership backing, and external supports that legitimize and routinize evidence-based addiction care.

## Key Points

**Question:**

What characteristics and supports enable effective champions to implement hospital-based opioid treatment (HBOT) in US hospitals?

**Findings:**

In this qualitative study of 31 hospital staff across 12 community hospitals, effective champions were perceived as respected insiders with institutional influence, persistence, and systems change skills. They built multidisciplinary teams, embedded HBOT into workflows, and overcame barriers through patient narratives. Champions were seen as most effective when supported by executive leadership, protected time, and external practice facilitation.

**Meaning:**

To expand HBOT, these findings suggest hospitals should invest in influential champions and provide leadership support, dedicated time, and external resources that can accelerate adoption of evidence-based addiction care.

## Teaser

This qualitative study evaluates characteristics and supports of effective clinical champions implementing hospital-based opioid treatment in US hospitals.

## Introduction

Opioid use disorder (OUD)–related hospitalizations have risen dramatically over the past 20 years and are associated with high morbidity and mortality.^1,2,3^ Yet life-saving evidence-based treatments, including medications for opioid use disorder (MOUD), are underutilized, with only 1 in 5 US residents with OUD receiving any MOUD.^4^ Hospitalization is a critical touchpoint and reachable moment for engaging patients in the OUD care continuum.^5,6,7^ Despite this, most hospitals do not offer high-quality OUD care, with only a minority of patients receiving MOUD and an even smaller proportion being linked to postdischarge care.^8,9,10^ Many hospital clinicians lack knowledge and comfort in prescribing MOUD.^10,11,12,13,14^

Hospital-based addiction care models,^15^ including addiction consult services (ACSs) and hospital-based opioid treatment (HBOT), improve substance use disorder and health outcomes by increasing medications for opioid and alcohol use disorder receipt, enhancing posthospital treatment engagement, and reducing readmissions and mortality.^16,17,18,19,20^ While ACSs consist of an interprofessional expert team, HBOT relies on generalists to integrate MOUD as part of routine hospital care.^15^ Compared with ACSs, HBOT typically uses existing staff, which may lead to unique implementation challenges.

Clinical champions for OUD care can play a key role in overcoming barriers and driving local culture change to increase the adoption of evidence-based OUD care.^21,22^ Research in general health care settings suggests that effective champions have strong communication, collaboration, and mentoring skills.^23,24^ In hospitals implementing ACSs, champions can help overcome financial challenges and institutional stigma by obtaining support through persistence and creativity.^25^ However, little is known about the specific attributes and supports needed for champions to effectively implement HBOT, which requires additional strategies to overcome unique barriers such as competing priorities and lack of OUD expertise.^14^

The Exemplar Hospital Initiation Trial to Enhance Treatment Engagement is a multisite implementation study supported by the National Institutes of Health Helping to End Addiction Long-Term Initiative and conducted in the National Institute on Drug Abuse Treatment Clinical Trials Network. This study was designed to support community hospitals in adopting HBOT to increase engagement in MOUD care after discharge by comparing low- and high-intensity implementation support and practice facilitation approaches.^26^ All participating hospitals received a best practices manual, video webinar series on HBOT topics, and hub team support for any questions, while hospitals randomized to the high-intensity group also received monthly practice facilitation (ie, tailored implementation support and addiction medicine expertise provided through coaching, site visits, and regular meetings) and partial funding for a local champion. Champions at community hospitals led HBOT implementation with support from local study hubs with expertise in HBOT. As the role of champions was central within this implementation trial, we explored hospital representatives’ perspectives on how champions influenced implementation using postimplementation qualitative interviews. The purpose of this qualitative study was to identify attributes of effective champions, contextual components of their effectiveness, and strategies used to overcome structural barriers. Findings can help hospital systems identify and support future champions to enhance their success.

## Methods

### Study Design, Setting, and Participants

From November 2023 through March 2024, we conducted postimplementation qualitative interviews with individuals engaged in HBOT implementation at 12 US community hospitals across 4 states (Massachusetts, Minnesota, New York, and Oregon) randomized to the high-intensity group of the trial.^26^ We used purposive sampling to recruit individuals with high levels of involvement in HBOT implementation, including the designated and emergent champions and hospital staff,^14,27^ followed by snowball sampling in which initial participants recommended additional participants to capture diverse perspectives and roles.^28,29^ We recruited participants until we reached sufficient information power,^30^ focusing on obtaining diverse perspectives on implementation. Participants provided verbal informed consent and were compensated $50. The Advarra, Inc, institutional review board approved study procedures. The study used the Consolidated Criteria for Reporting Qualitative Research (COREQ) reporting guideline and was registered at clinicaltrials.gov at NCT04921787

### Data Collection

A lead qualitative investigator (A.R.B.) trained and supervised interviewers (research staff not involved in implementation) to use a semistructured interview guide informed by the Reach, Effectiveness, Adoption, Implementation, and Maintenance (RE-AIM) framework,^31,32^ the protocol’s implementation research logic model,^26^ and findings from baseline qualitative interviews.^14,27^ The guide was critically reviewed by hub investigators and local hub team members for face validity, with minor refinements made to improve clarity. Open-ended questions and detailed probes explored hospitals’ HBOT implementation, including questions on clinical champions (eg, “How effective do you think the champion has been in your hospital?” and “How could the project have better supported the champion in your hospital?”). A truncated interview guide is available in eMethods in Supplement 1. Interviews were conducted virtually (eg, videoconferencing), lasted approximately 45 to 60 minutes, and were audio recorded and professionally transcribed, with transcripts reviewed for accuracy and deidentification following a structured protocol.^33^

### Data Analysis

We employed a combined deductive and inductive approach, using a collaborative codebook development process in which the lead expert qualitative investigator (A.R.B.) and 6 research (R.S., A.P., C.K., and E.O.) staff developed preliminary codes based on interview questions, RE-AIM, relevant literature, and emergent observations from the team.^34,35^ We independently tested preliminary codes on 3 transcripts, discussed discrepancies, and refined the codebook through 3 additional rounds of testing until reaching consensus on a final codebook that included a specific code for champions. Four analysts (C.K., A.P., and E.O.) then independently applied final codes to transcripts using NVivo 4 (Lumivero), meeting weekly to monitor coding consistency and discuss emergent findings.^36^ We then used the framework method to summarize coded data at the participant level, extract exemplar quotes, and write higher-level memos interpreting data within each code.^37^ The lead author (L.P.) then conducted in-depth, thematic analysis of coded data and memos pertaining to champions.

We defined effective champions as those perceived by hospital staff and stakeholders to be effective in driving HBOT implementation efforts. While future analyses will focus on determining whether and why hospitals achieved increases in HBOT delivery, our study team noted emerging consensus across the study team and participants that champions played an important role in HBOT implementation that warranted in-depth exploration and description.

## Results

We interviewed 31 hospital staff (15 physicians, 5 executives, 5 pharmacists, 2 nurse practitioners, 2 social workers, 1 nurse, 1 addiction counselor) across 12 community hospitals (10 urban and 2 rural), with 2 to 3 participants interviewed at each site. Champions were self-identified and included 12 clinical champions, 4 executive leadership champions, 1 outpatient addiction program administrator, and 1 community emergency medical services champion; 3 participants reported addiction expertise. Hospitals ranged from 50 to 99 beds (1 hospital), 100 to 299 beds (2 hospitals), 300 to 499 beds (6 hospitals), to 500 or more beds (3 hospitals).

We identified 3 major themes: (1) effective champions were influential within their organizations, strongly committed, and determined to drive change; (2) effective champions built coalitions of multidisciplinary hospital staff and used patient narratives to overcome barriers and embed HBOT into standard practices; and (3) executive sponsorship and practice facilitation supported champions to overcome structural stigma and institutional barriers. These themes and supporting subthemes are shown with illustrative quotes in the Box. The Figure demonstrates the central role of champions amidst other factors supporting HBOT implementation.

Box. **Champion Themes and Illustrative Quotes**

**1. Champion Characteristics**

**1.1 Respected Hospital “Insiders”**

“He’s a guy who has a lot of weight in the hospital. The sort of person people listen to.” (Physician)
“The person was an insider to the hospitalists, who is our main target audience for behavior change. So, she was welcomed as an insider and respected as an addiction specialist, an addiction researcher. She was able to communicate, connect, and influence hospitalists.” (Executive sponsor)

**1.2 Determined and Pragmatic**

“I don’t think we would have made it as far as we did had it not been somebody that was as passionate about the project, had they not wanted to see this to fruition. Anybody else could have very easily just been like, ‘This is the status quo. I’ve checked the box, and we’ve done the best we could.’” (Social worker)
“I think my approach from the beginning was we can’t let perfect be the enemy of good. We need to get something organized and systematic. We need to get it going and pilot it and run some cycles on it. We didn’t get too bogged down in trying to make it the world’s greatest, most perfect plan of all time.” (Physician, champion)

**1.3 Systems Change Skills Over Addiction Expertise**

“Learning how to prescribe medications is important. But the key to addiction is building a system that supports people and prescribers. And to have a study where we were supported looking at our protocols how to change things at a systems level was just very impactful. I think the changes that we’ve done over the past two years, whether it be culture change, order sets, or how we involve pharmacy, had far greater impact than had it just focused on teaching people how to prescribe meds.” (Physician, champion)
“I’m not an addiction medicine physician. My expertise was not clinical expertise. But maybe that was actually an advantage because I wasn’t speaking to my colleagues from a place of ‘I know everything there is to know about opioid use disorder.’ It was more, this is something I’m seeing as a hospitalist, and from what I’ve learned, this is fairly straightforward, and we can make a big difference in the inpatient setting.” (Physician, champion)

**2. Champion Actions**

**2.1. Built Effective Multidisciplinary Teams**

“My role was to get a team of people together who would be influential, and get things done, and multidisciplinary…so discharge planning, informatics, pharmacy, social work, hospitalist, and medical—other medical staff.” (Physician, champion)
“Things that really set us up at the beginning to do well—we included as many stakeholders as we could think of. We had pharmacy involvement from the beginning, nursing, CD counselors, and case managers were involved.” (Physician)

**2.2. Leveraged Patient Narratives Combined With Data**

“She [community liaison] has all this artwork from either people who were impacted or had OUD. That was really impactful. We’re working with that community liaison, that director, and the C-suite to bring that artwork into our main lobby.” (Pharmacist)
“Once we had a couple people who benefited from this, [it] becomes very rewarding and motivating for the entire team when they hear those stories.” (Executive sponsor)

**2.3. Created Durable Systems to Institutionalize HBOT**

“Our work over the last two years has significantly improved that flow with a clear treatment pathway that is evidence-based… it’s been helpful to have an agreement that this is an important issue that our hospital system needed to address.” (Physician, champion)
“The biggest key to sustain it for us will be making sure we continue to educate new people and making sure that we have the standard tools [workflows, order sets] to be able to keep it sustainable.” (Executive sponsor)
“I felt like our biggest focus was setting an infrastructure together. Keeping the culture and keeping the expectation, that was a big hurdle…So, that’s gonna be the biggest hurdle is keeping it a priority as other things seem to take precedence.” (Physician, champion)

**3. Champion Supports**

**3.1. Executive Sponsorship and Champion Protected Time**

“My role is to facilitate development of programs and clinical services in the hospital and addressing care gaps. It was good for this project to have my role as in charge of the quality pillar to be the champion for this. There was never an issue with administrative support or being able to utilize other group members’ time. It’s within my purview to be able to ask for those resources, that’s one benefit of having somebody that sits at the [VP] level being part of this, there’s automatic buy-in from the administration.” (Executive sponsor)
“Give the champion time and support and have committed hospital leadership to work on mitigating and ameliorating obstacles and minimizing headwinds because there are a lot of obstacles besides just dropping an order set or telling people what’s expected and what’s best practice.” (Executive sponsor)

**3.2. Lack of Leadership Support Limited Champion Effectiveness**

“There was going to be sort of a dyad partnership, and then he [medical director] unexpectedly retired, and it was just like the project died. The new medical director didn’t provide the project champion with any support because she didn’t agree that it should be a hospitalist-led program. And so, the project champion floundered in the role of project champion once she lost her dyad partnership.” (Nurse manager)
“I don’t know that I have been that effective. Without somebody in leadership to say, ‘hey, we want to do this work,’ I didn’t have any power to make it happen. I didn’t have any power to tie any kind of quality incentives to the work or to audit and see if people were doing the work. It was just suggestion, but if people chose not to participate there was nobody that was going to step in.” (Nurse practitioner, champion)

**3.3. Practice Facilitation Provided Guidance and Expertise**

“So, that [involvement in the study] gave validation to the idea of having this in our own hospital when you have leading healthcare institutions also doing this.” (Nurse practitioner, champion)
“The majority of changes are attributed to the research with [hub]. Having those colleagues on the backend who we could go to for questions, and who would send out different articles so that we could learn how to treat these patients better.” (Pharmacist)
“The reason it’s happening is because HBOT kept us focused on it. It would’ve been very easy for me to push it aside because I have so many other system-wide things. Maybe the best thing was just someone following up every month.” (Executive sponsor)

**Figure.**
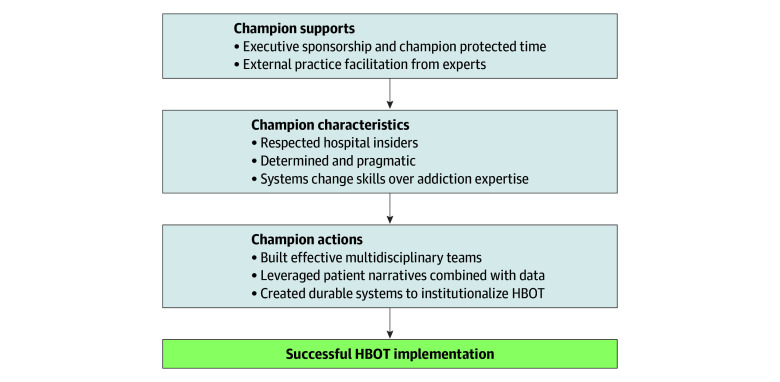
The Role of Champions and Other Factors Supporting Hospital-Based Opioid Treatment (HBOT) Implementation

### Champion Characteristics

#### Position Within the Hospital

Champions had diverse clinical roles, including hospitalists, emergency physicians, addiction medicine specialists, and pharmacists. However, champions’ job titles were perceived to be less important than being respected hospital “insiders” with established professional relationships. For example, one champion had “been at the institution for many years, and built relationships with most hospitalists over time,” earning their respect. This insider status, coupled with credibility and relationships, allowed them to directly engage staff to more effectively “communicate, connect, and influence” their colleagues.

#### Characteristics

Effective champions were deeply committed to improving OUD care and unwilling to accept the “status quo.” They demonstrated persistence in the face of institutional barriers, consistently showing up and working to make progress. “Not [letting the] perfect be the enemy of good,” these champions prioritized progress and building systems that could be improved over time. This pragmatic mindset, combined with persistence, helped champions push forward despite system barriers.

#### Approach to Systems Change

While some champions had clinical expertise in addiction medicine, many did not. Champions without addiction expertise were still viewed as highly effective when they were skilled in leading systems-level change. One champion noted that it was “actually an advantage” to be able to speak to colleagues as a nonexpert. Effective champions leveraged outside addiction medicine expertise and practice facilitation for addiction-specific knowledge, while drawing on their own expertise in institutional processes and workflows to “create a system that supports [patients] and prescribers.”

### Champion Actions

#### Team Building

Effective champions recognized that successful HBOT implementation required more than individual effort and depended on capable, multidisciplinary teams. Champions viewed their role as facilitators of collaboration, stating their responsibility was to “get a team together, meet, [and] problem solve.” Champions intentionally recruited team members from key hospital departments—including pharmacy, social work, discharge planning, informatics, hospital medicine, and hospital executives—who brought varied, discipline-specific knowledge, were respected, and could influence change. Participants described these team members as people who could “get things done,” enabling champions to create workflows and lead systems change more efficiently.

#### Obtaining Buy-In

Many participants described champions’ challenges obtaining buy-in from hospital executive leadership as a major barrier to HBOT implementation. In response, champions used creative strategies to build coalitions of influential hospital staff and community representatives who could help promote the urgency and legitimacy of HBOT. One champion hosted a gathering at her home, bringing together executives, hospital staff, and community members to share personal and patient stories—an approach that successfully motivated leadership to support HBOT. Another champion created an art exhibit featuring work by individuals affected by OUD, which hospital staff described as “incredibly impactful to see…how that emotion is displayed in art form.” Champions also used patient stories and early successes to maintain momentum and inspire others. These emotionally resonant strategies helped humanize the need for HBOT, shifting leadership perspectives and priorities.

#### Creating Systems

While champions used a range of strategies to implement HBOT, the most effective champions focused on creating standardized processes to support sustainability, including developing treatment pathways, electronic medical record order sets, and other “standard tools” that embedded HBOT into routine workflows. Hospital staff emphasized that these broader structural interventions had a far greater impact than brief didactics that “just [teach] people how to prescribe meds.”

They emphasized that successful HBOT implementation required more than tools—it required a cultural shift. By setting the “expectation” that HBOT was part of standard care and securing buy-in from hospitalists and multidisciplinary staff, champions fostered a broader change in hospital “attitudes” toward patients with OUD. Some champions further strengthened sustainability by embedding dedicated roles, such as a substance use navigator, into the hospital system to provide ongoing support for HBOT delivery. These efforts encouraged continued HBOT even amid changes in staffing, priorities, or leadership.

### Champion Supports

#### Executive Sponsorship

While effective champions strove to embed HBOT into routine practice, these efforts were strengthened—or sometimes constrained—by the level of leadership support. Executive leadership, defined here as senior hospital decision-makers with the authority to allocate resources, set strategic priorities, and influence organizational culture (eg, Chief Medical Officers [CMO] or department heads), played a critical role in facilitating implementation. Executive sponsorship lent credibility to HBOT efforts, helping champions overcome practitioner and staff resistance. Executive leadership allocated administrative and other support, enabling smoother implementation. One hospital executive emphasized the importance of supporting the champion, stating: “Don’t underestimate the importance of the champion…give [them] time and support to do what they need to do. And have committed hospital leadership to work on mitigating and ameliorating obstacles and minimizing headwinds.”

Importantly, both hospital staff and champions emphasized that protecting champion time was one of the most meaningful ways hospital leaders could provide support. Several champions noted that after the formal study period ended, it was difficult to find time for HBOT since they were “basically doing it in [their] spare time” and “[paying] themselves through passion.” They underscored the need for dedicated time to sustain HBOT beyond initial implementation.

#### Lack of Leadership Support

Some champions reported feeling like they “didn’t have any power to make it happen,” underscoring the difficulty of making systems change without executive sponsorship. Hospital staff observed that some inexperienced champions “floundered in the role” without the support of someone who understood how to “get things accomplished within our system.” In the absence of executive leadership support, champions faced unanticipated “roadblocks” that made developing new workflows and obtaining institutional buy-in more difficult.

These challenges were further compounded by negative attitudes toward patients with OUD. As one champion stated, “people who have negative views sometimes stand out more…there are still people who are not supportive of medications for opioid use disorder.” Some champions had difficulty overcoming vocal critics of HBOT, especially when these critics had influence or felt that specialty training was necessary to deliver OUD care.

#### Practice Facilitation

Champions and hospital staff consistently highlighted the value of HBOT practice facilitation from national experts in supporting implementation by maintaining accountability and keeping teams “focused,” which catalyzed change and provided structure. While practice facilitation as a strategy does not necessarily require specialty clinical expertise, HBOT implementation teams appreciated that in this study, facilitators offered clinical expertise in addiction medicine, helping them feel more confident in promoting and delivering HBOT. Practice facilitation also served as “validation” by providing reassurance and encouragement from external experts. Learning collaboratives set up through practice facilitation allowed cross-site teams to learn from one another’s successes and setbacks, legitimizing HBOT efforts.

## Discussion

In this national qualitative study of 12 community hospitals, effective HBOT champions were described as respected “insiders” who built coalitions, demonstrated a practical approach toward embedding care into standard workflows, and drove culture change (shifting hospital norms and attitudes toward OUD care). Most champions did not have addiction medicine expertise; however, experience in systems change, hospital leadership support, and external practice facilitation from national addiction experts enabled HBOT implementation despite this lack of specialty training.

Although prior work describes the importance of clinical champions in promoting evidence-based practices, limited literature details the attributes that make champions effective at improving addiction care in hospitals.^22,23,38,39^ Unlike prior studies of ACS models or champions in general, this study highlights how HBOT champions operate in settings where generalists must overcome stigma and competing priorities with limited addiction expertise. HBOT implementation may be particularly challenging compared with ACS because HBOT relies on generalists who are responsible for patients’ overall clinical care, have limited bandwidth, competing priorities, and often lack knowledge and confidence in delivering OUD care—barriers that are difficult to overcome without institutional culture change and structural supports.^15^ Without dedicated resources and time, champions may lack the capacity and expertise to provide education and training, develop new workflows, and establish postdischarge community partnerships, all of which are important components of HBOT. Existing research suggests that a combination of personal attributes—such as influence, persistence, and strong communication skills—alongside external and institutional supports, are critical for champions to drive meaningful change within health care systems.^24,25,40^ Our findings build on this work by identifying how these attributes operate in the context of HBOT implementation, where champions succeeded through systems thinking and relational leadership.

Consistent with prior work,^22,25^ our study reinforces the importance of hospital leadership buy-in and external support. Executive leadership support, especially from senior hospital decision-makers (eg, CMO), legitimized HBOT, encouraged staff engagement, and reduced stigma—especially when paired with protected time for champions. Effective champions used powerful storytelling—patient narratives and visual art—to communicate the importance of HBOT and overcome leadership reluctance. In contrast, less effective champions, especially those lacking systems change experience, often struggled to make progress in the absence of institutional support. Practice facilitation was also perceived as supporting HBOT implementation, especially for champions who lacked addiction expertise or experience with systems change. Facilitators provided structured guidance, accountability, clinical education, and cross-site learning opportunities to help maintain momentum and further lend credibility to HBOT efforts.

Our results suggest that health systems seeking to expand HBOT should prioritize individuals with institutional influence and systems thinking skills while also investing in dedicated protected time and leadership endorsement. For policymakers, our findings underscore the value of investing in practical support structures, including external facilitation supports and technical assistance. Policies that incentivize hospital-based addition care and support the development of implementation leadership skills in the workforce could accelerate adoption of HBOT, particularly in resource-limited hospitals. As hospitals respond to the ongoing overdose crisis, targeted policies that strengthen local champions and the systems that support them will be critical for expanding access to life-saving treatment.

### Limitations

Our study has important limitations. Champions were supported with funding for protected time as well as structured practice facilitation from national experts, which may limit the generalizability of findings to health care settings without similar external support. Nonetheless, our results offer key insights into the attributes and supports that facilitate successful champion-driven implementation. Additionally, because we defined effectiveness based on hospital staff perceptions rather than objective outcomes, it remains unknown whether perceived effective champions ultimately increased HBOT delivery. If perceived effectiveness does not correlate with higher HBOT uptake, further investigation will be needed to understand why. Future analyses will examine quantitative differences in HBOT delivery across hospitals and identify contextual and implementation factors associated with those outcomes. These findings on perceived champion effectiveness provide important context for interpreting future quantitative results.

## Conclusions

This multisite qualitative study underscores the vital role of clinical champions in expanding hospital-based opioid care. Champions were perceived as respected insiders with persistence, institutional influence, and skills in driving systems change. They built effective teams, integrated HBOT into routine workflows, and overcame structural barriers, particularly when supported by hospital leadership and external facilitators. To ensure lasting expansion of HBOT, hospitals should invest in champions, protected time, leadership backing, and external supports that legitimize and routinize evidence-based addiction care.

## Notes

Supplement 1. eAppendix. Survey Instrument

Supplement 2. Data Sharing Statement
