# Supplementary material for: Perspectives on Clinical Champions Implementing Hospital-Based Opioid Treatment in US Hospitals
Source: JAMA Netw Open. 2026 Mar 3;9(3):e260446. doi: 10.1001/jamanetworkopen.2026.0446 (PMC12958086; doi:10.1001/jamanetworkopen.2026.0446)
Supplement: Supplement 1. — eAppendix. Survey Instrument [file jamanetwopen-e260446-s001.pdf]

Supplemental Online Content

Peng L, Goff A, Patten A, et al. Perspectives on clinical champions implementing hospital-based opioid treatment in US hospitals. *JAMA Netw Open*. 2026;9(3);e260446. doi:10.1001/jamanetworkopen.2026.0446

eAppendix. Survey Instrument

This supplemental material has been provided by the authors to give readers additional information about their work.

© 2026 Peng L et al. *JAMA Network Open*.

EXHIT ENTRE: Exemplar Hospital Initiation Trial to Enhance Treatment Engagement – Hospital Based Opioid Treatment Intervention Implementation Study: NIDA CTN 0098B

Post-Implementation (Month 24) Qualitative Truncated Interview Guide (*Champion-related items only*)

Section 1. Individual Role and Opioids

1. **To get started, can you briefly describe your role related to opioid use disorder (OUD) treatment?**

   Champion: Do you consider yourself to the "champion" for this work?

   If Yes: Was this "champion" role a new role for you within the past 2 years?

Section 3. Experiences with the Project

2. **Thank you. Now I'd like to ask you some more specific questions about this project, which was designed to implement hospital-based OUD treatment, or "HBOT" for short. What role did you play in this project?**

3. **Tell me about your interactions with the [HUB NAME] team facilitators.**
   a. How did the facilitators assist implementing HBOT?
   b. Was there anything the facilitators could have done to better support implementing HBOT?

4. **CHAMPIONS: Having a local champion can help support HBOT implementation. As an identified champion, how has this project supported you in this role?**
   a. How could this project have better supported you as a champion?
   b. How effective do you think you have been as a champion? Why?

5. **NON-CHAMPIONS: Having a local champion can help support HBOT implementation. How do you think this project supported the champion in your hospital?**
   a. How could this project have better supported the champion in your hospital?
   b. How effective do you think the champion has been in your hospital? Why?

6. **How has this project impacted staff attitudes or feelings towards patients with OUD?**

   Optional probes (if elaboration seems needed):
   How can you tell?
   Can you provide some examples?

7. **How has this project impacted leadership attitudes towards OUD care?**

© 2026 Peng L et al. *JAMA Network Open*.

Optional probes (if elaboration seems needed):
How can you tell?
Can you provide some examples?

Section 4. Additional Context & Reflections

8. **For the following questions, please think about your overall experience with this HBOT-implementation project over the past 2 years:**

   a. What was the most challenging or discouraging thing about the experience?

   b. What was the most rewarding thing about the experience?

   c. What was the most unexpected thing about the experience?

9. **Do you think your hospital will maintain, increase, or decrease its current level of inpatient MOUD prescribing?**

   a. What could hinder efforts to maintain HBOT as part of standard clinical care?

   b. What kinds of specific plans, if any, does your hospital have to maintain HBOT?

   c. CHAMPION: How do you envision your role (and time allotted to it) moving forward?

What advice would you give to future implementers of initiatives to improve hospital-based OUD treatment?

© 2026 Peng L et al. *JAMA Network Open*.
