# Supplementary material for: Perspectives on Clinical Champions Implementing Hospital-Based Opioid Treatment in US Hospitals
Source: JAMA Netw Open. 2026 Mar 3;9(3):e260446. doi: 10.1001/jamanetworkopen.2026.0446 (PMC12958086; doi:10.1001/jamanetworkopen.2026.0446)
Supplement: Supplement 2. — Data Sharing Statement [file jamanetwopen-e260446-s002.pdf]

Data Sharing Statement

Peng. Perspectives on Clinical Champions Implementing Hospital-Based Opioid Treatment in US Hospitals. *JAMA Netw Open*. Published March 03, 2026.
doi:10.1001/jamanetworkopen.2026.0446

Data
Data available: No

Additional Information
Explanation for why data not available: No qualitative data are available for sharing because the data consist of interview transcripts that cannot be sufficiently de-identified.
